# Supplementary material for: Molecular definition of multiple sites of antibody inhibition of malaria transmission-blocking vaccine antigen Pfs25
Source: Nat Commun. 2017 Nov 16;8:1568. doi: 10.1038/s41467-017-01924-3 (PMC5691035; doi:10.1038/s41467-017-01924-3)
Supplement: Supplementary file 1 — Supplementary Information [file 41467_2017_1924_MOESM1_ESM.pdf]

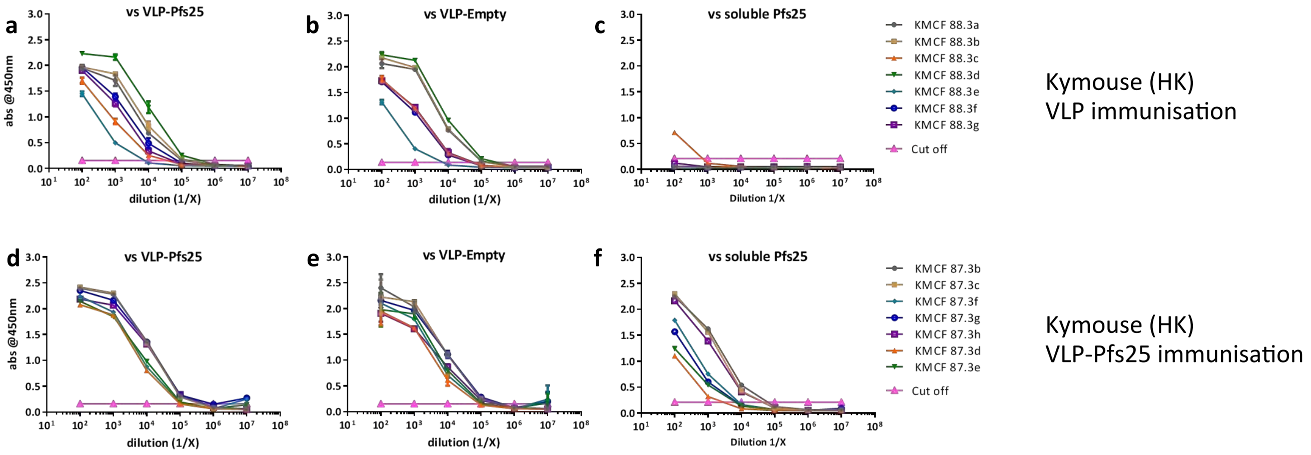

Supplementary Figure 1. Serum polyclonal titers measured by ELISA for Kymice engineered to express the full set of human immunoglobulin variable, diversity and joining region gene segments for the immunoglobulin heavy chain and the kappa light chain variable and joining region gene segments (HK Kymice). Serum polyclonal antibody titer for each immunized mouse (KMFC numbers) were determined by limiting dilution ELISA against plate bound VLP-Pfs25 (**A** and **D**), VLP (VLP-Empty) (**B** and **E**) and soluble Pfs25 protein (**C** and **F**). Kymice were immunized with either naked VLP (VLP-Empty (panels **A** to **C**) or VLP-Pfs25 (panels **D** to **F**). All mice raised a polyclonal antibody response to the VLP regardless of the presence or absence of the additional Pfs25 antigen (**B** and **E**), but only VLP-Pfs25 mice raised a polyclonal response to Pfs25 (Panel A and D compared to panels C and F).

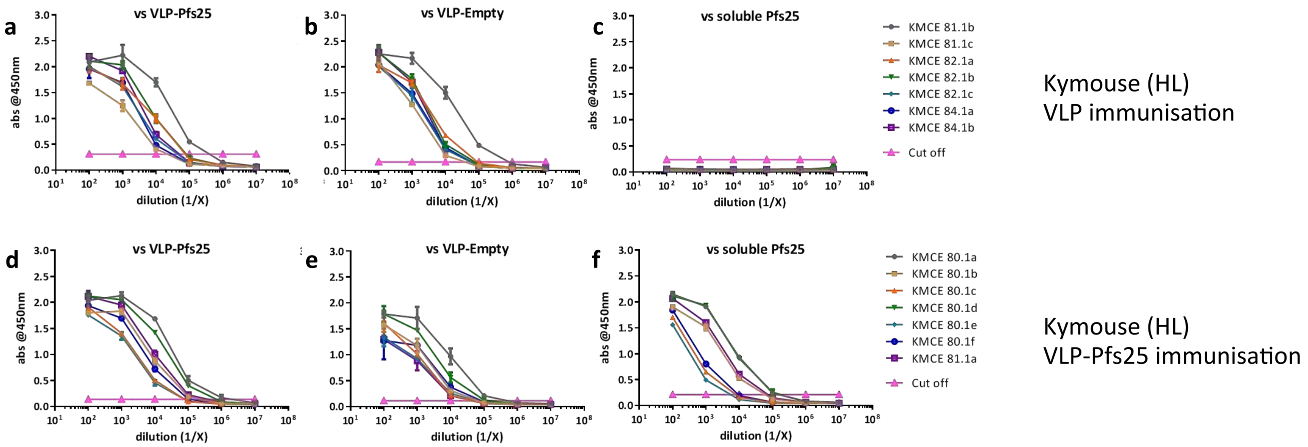

Supplementary Figure 2. Serum polyclonal titers measured by ELISA for Kymice engineered to express the full set of human immunoglobulin variable, diversity and joining region gene segments for the immunoglobulin heavy chain and the lambda light chain variable and joining region gene segments (HL Kymice). Serum polyclonal antibody titer for each immunized mouse (KMFC numbers) were determined by limiting dilution ELISA against plate bound VLP-Pfs25 (**A** and **D**), VLP (VLP-Empty) (**B** and **E**) and soluble Pfs25 protein (**C** and **F**). Kymice were immunized with either naked VLP (VLP-Empty (panels **A** to **C**) or VLP-Pfs25 (panels **D** to **F**). All mice raised a polyclonal antibody response to the VLP regardless of the presence or absence of the additional Pfs25 antigen (**B** and **E**), but only VLP-Pfs25 mice raised a polyclonal response to Pfs25 (Panel A and D compared to panels C and F).

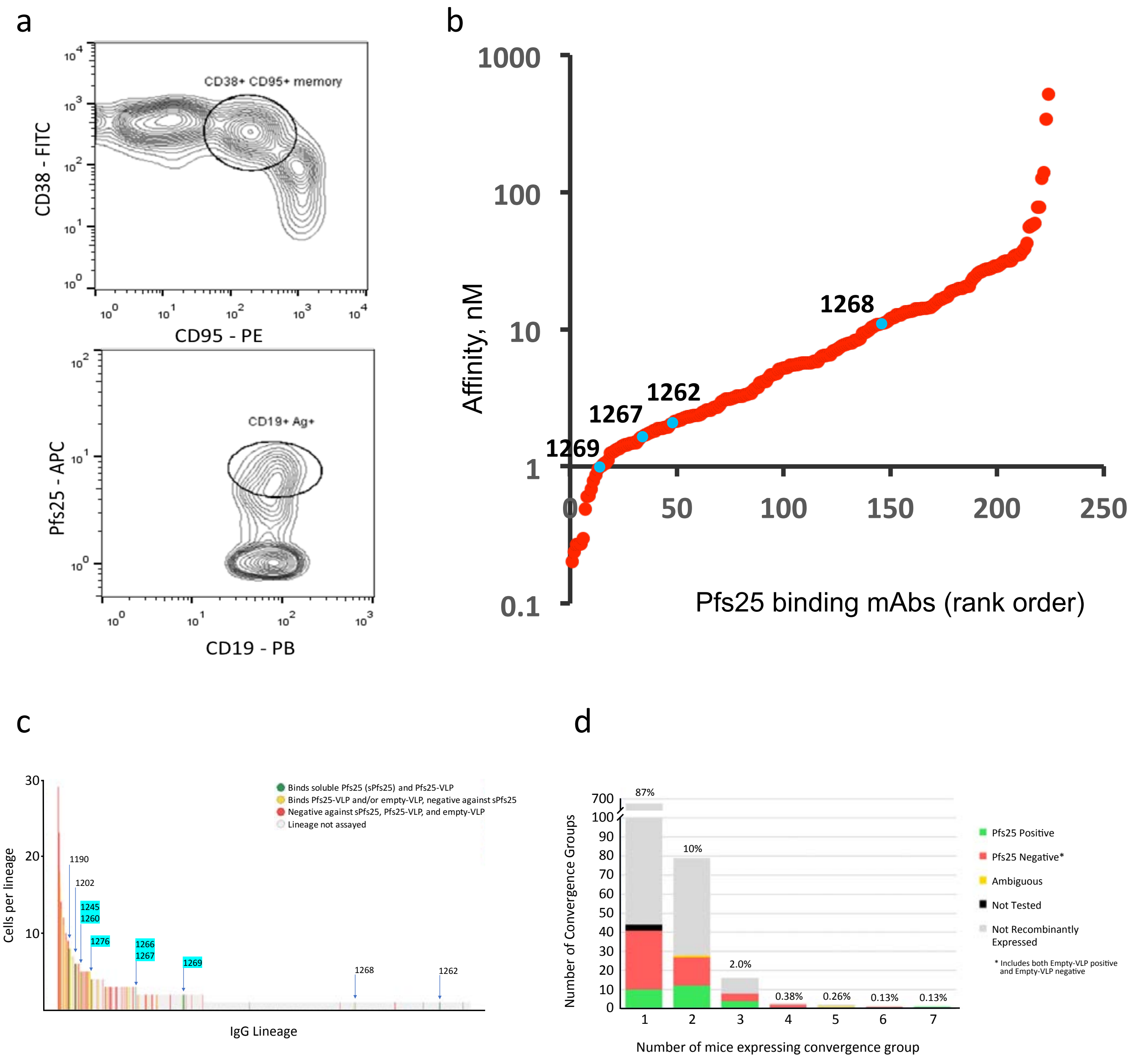

Supplementary Figure 3. Gating strategy for antigen specific memory/GC B cells and IgG lineage analysis. (A) The flow cytometric contour plot displaying the gating of CD38+ (FITC) CD95+ (PE) memory cell population after the exclusion of IgM^{+} and IgD^{+} B cells. Single memory B cells positive for $CD19^{+}$ (Pacific Blue) and antigen positive (Pfs25-APC) were then sorted into separate wells in a 96-well plate. **(B)** The binding affinity (nM) of 225 Pfs25 antigen binding antibodies assayed by SPR to surface immobilized Pfs25 antigen with the positions of antibodies 1262, 1267, 1268 and 1269 indicated in blue. Antibodies 1276, 1260 and 1266 were not expressed at sufficient quantity in this high-throughput assay format to allow SPR measurements. **(C)** Summary of ELISA results for plasmablast-derived and memory B cell-derived sequences according to their Ig lineage and the number of cells per lineage. Lineages on the x-axis are based on natively paired HC+LC sequences. Labels in blue highlight denote lineages for which both plasmablasts and memory B cells were observed. Other labels denote

lineages for which only plasmablasts or only memory B cells were observed. **(D)** Summary of ELISA results for plasmablast-derived and memory B cell-derived sequences according to the number of Kymice that share the same lineage. Lineages that appear the same, but that originate from different Kymice, together make a convergence group. The number of convergence groups is plotted against the number of Kymice in a convergence group (e.g. 87 % of IgG observed lineages occur in a single Kymouse, while 10 % of IgG lineages occur in two separate Kymice).

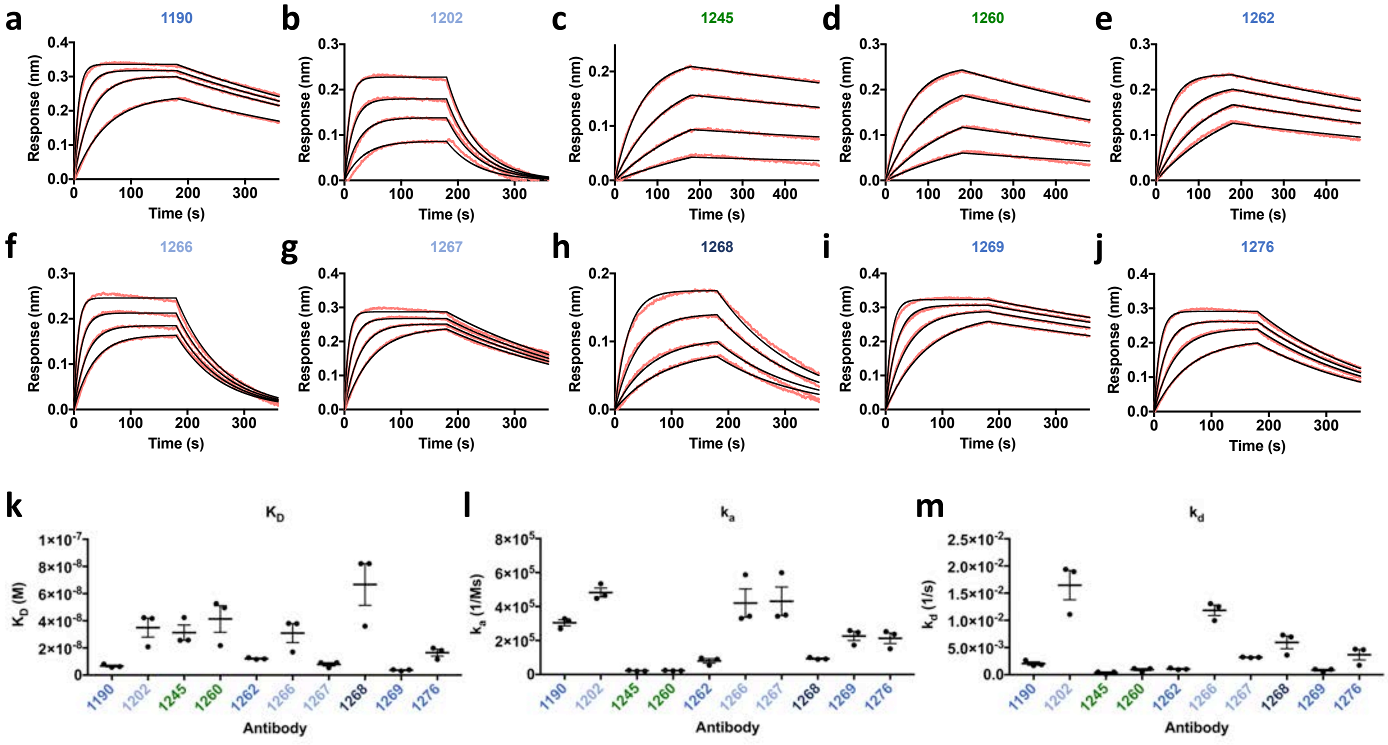

Supplementary Figure 4. Binding kinetics of anti-Pfs25 mAbs. (A-J) Representative sensorgrams (salmon) and 1:1 model best fits (black) for Fab binding to Pfs25, determined by biolayer interferometry. A 2-fold dilution series was used beginning at the following concentrations **(A)** 1190 – 400 nM, **(B)** 1202 – 200 nM, **(C)** 1245 – 800 nM, **(D)** 1260 – 800 nM, **(E)** 1262 – 400 nM, **(F)** 1266 – 400 nM, **(G)** 1267 – 400 nM, **(H)** 1268 – 400 nM, **(I)** 1269 – 400 nM, **(J)** 1276 – 400 nM. The **(K)** K_D, **(L)** k_a and **(M)** k_d for each antibody. Three independent measurements were performed (black circles) and mean and standard error of the mean (SEM) are shown. Antibodies names are colored by their respective bin from Fig. 2. If antibodies belong to two or more bins, then only one was selected for coloring.

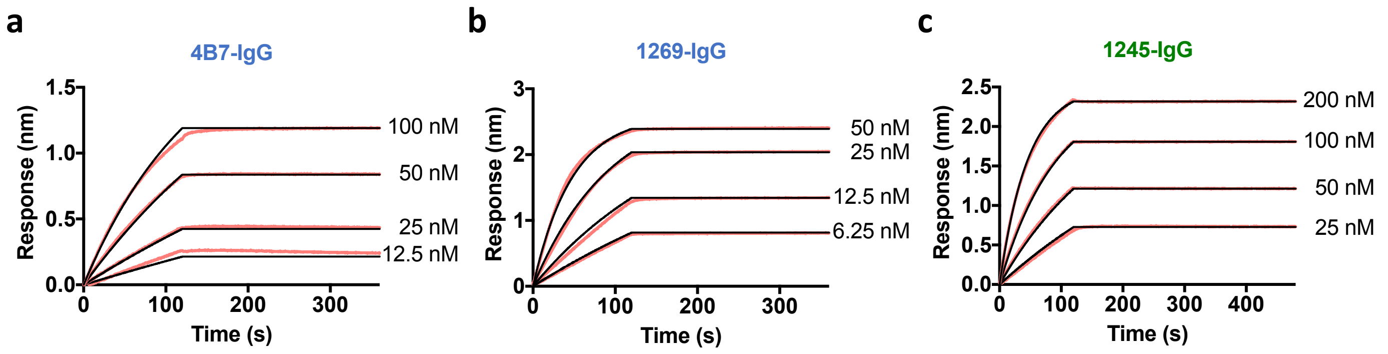

Supplementary Figure 5. Binding kinetics of site 1 and site 2 mAbs. Representative sensograms (salmon) and 1:1 model best fits (black) for **(A)** 4B7-IgG (control), **(B)** 1269-IgG and **(C)** 1245-IgG binding to Pfs25, determined by bioloyer interferometry.

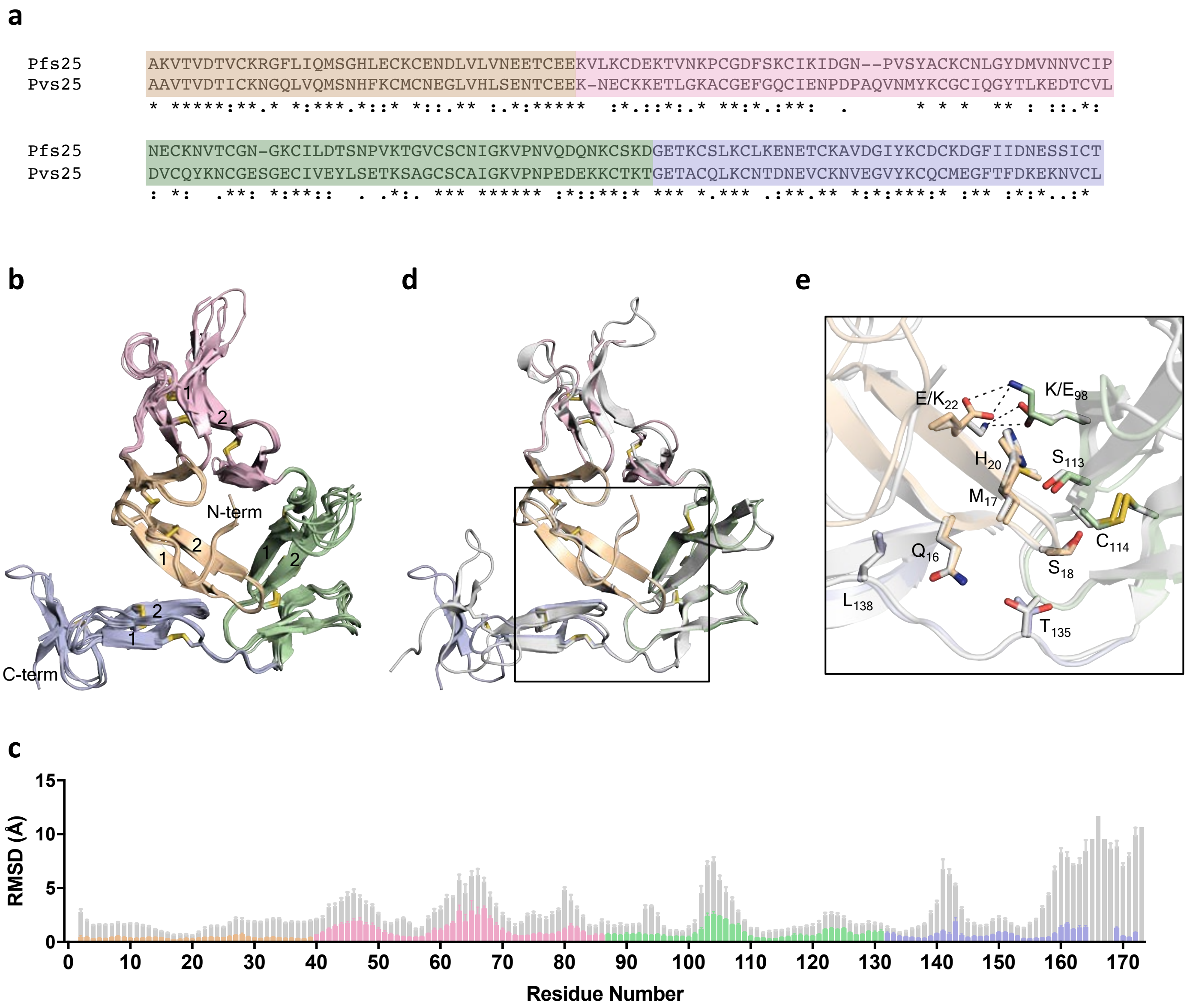

Supplementary Figure 6. Comparison of Pfs25 crystal structures. (A) Sequence alignment of Pfs25 and Pvs25 generated using Clustal Omega[1]. The sequence corresponding to EGF-like domains 1-4 are colored in wheat, pink, green and blue, respectively. **(B)** Superposition of the six Pfs25 crystal structures shown as cartoon representation, with EGF-like domains colored as in (A). Disulfide bonds are represented as sticks. **(C)** The RMSD between the six Pfs25 crystal structures (main-chain atoms) is shown with bars colored according to EGF-like domain as in (A). The RMSD between the six Pfs25 crystal structures and the Pvs25 crystal structure is shown with bars colored in grey (main-chain atoms, PDB 1Z1Y). Mean and SEM are reported. Calculated using MOE 2015.10[2]. **(D)** Superposition of the Pfs25 structure (complexed with 1276) and Pvs25 (PDB 1Z1Y) colored in grey[3]. **(E)** Inset from figure (D), detailing residues conserved between Pfs25 and Pvs25 that contribute interactions that promote the triangular arrangement.

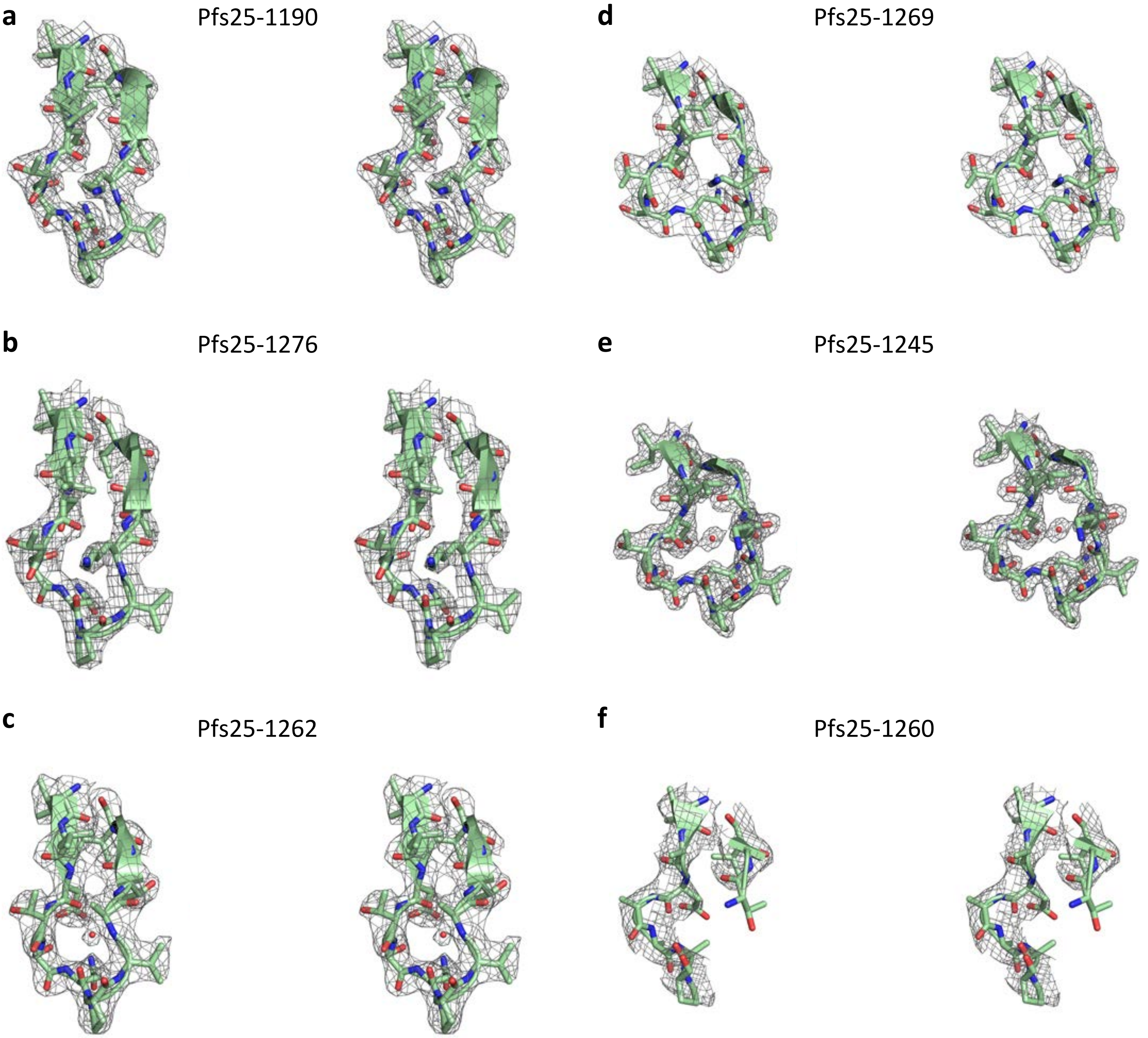

Supplementary Figure 7. Stereo view of the conformational differences in the 4B7 loop region. Stereo view of the 2mFo-DFc electron density map contoured at 1.0 σ of the 4B7 loop, residues 100 to 111 of Pfs25 bound to **(A)** 1190, **(B)** 1276, **(C)** 1262, **(D)** 1269, **(E)** 1245 and **(F)** 1260. The 4B7 loop displays conformational flexibility between the crystal structures.

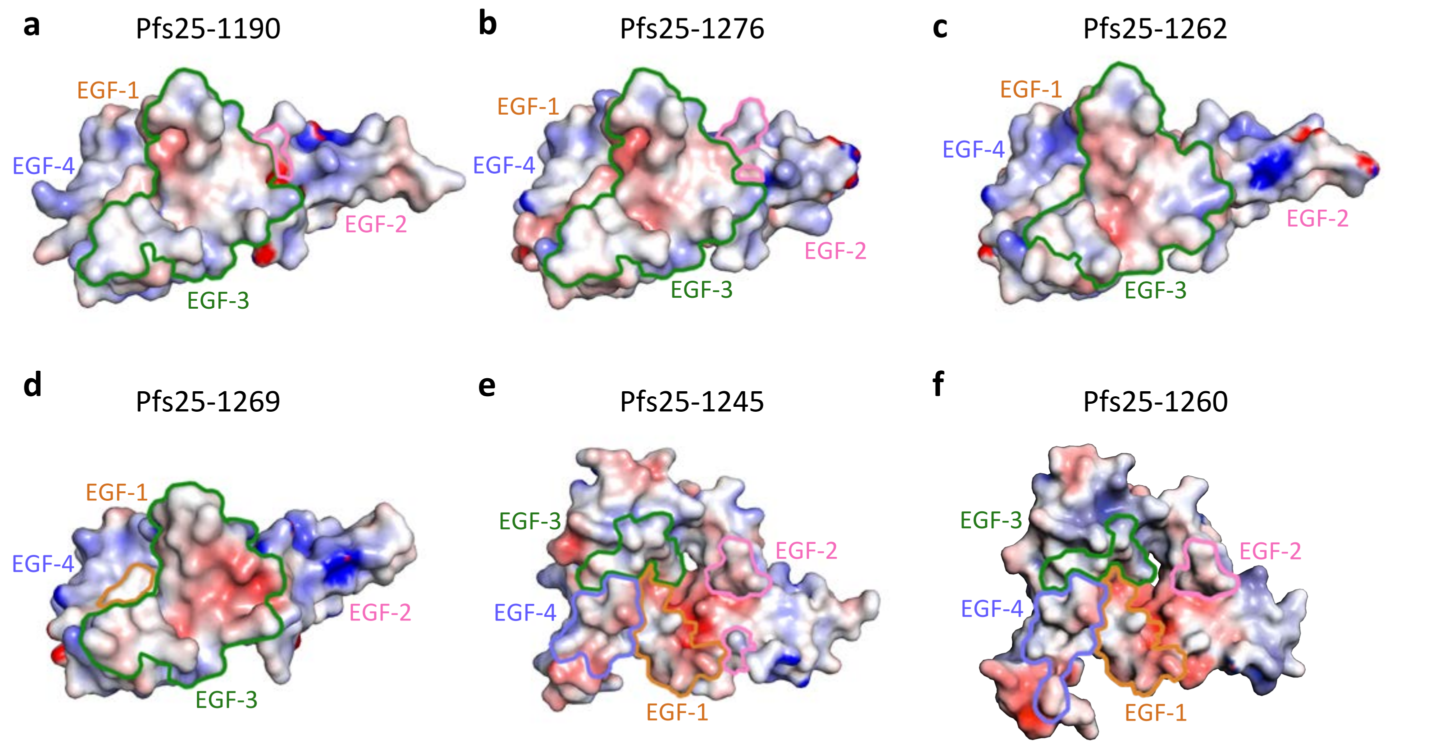

Supplementary Figure 8. Surface electrostatics of Pfs25 antibody recognition sites. The solvent accessible electrostatic potential is shown for **(A)** Pfs25-1190, **(B)** Pfs25-1276, **(C)** Pfs25-1262, **(D)** Pfs25-1269, **(E)** Pfs25-1245 and **(F)** Pfs25-1260. Electrostatic calculations were performed using APBS (± 5 *kT/e*)[4]. Antibody recognition sites are outlined and colored according to EGF-like domain (wheat – EGF-like domain 1, pink – EGF-like domain 2, green – EGF-like domain 3, blue – EGF-like domain 4).

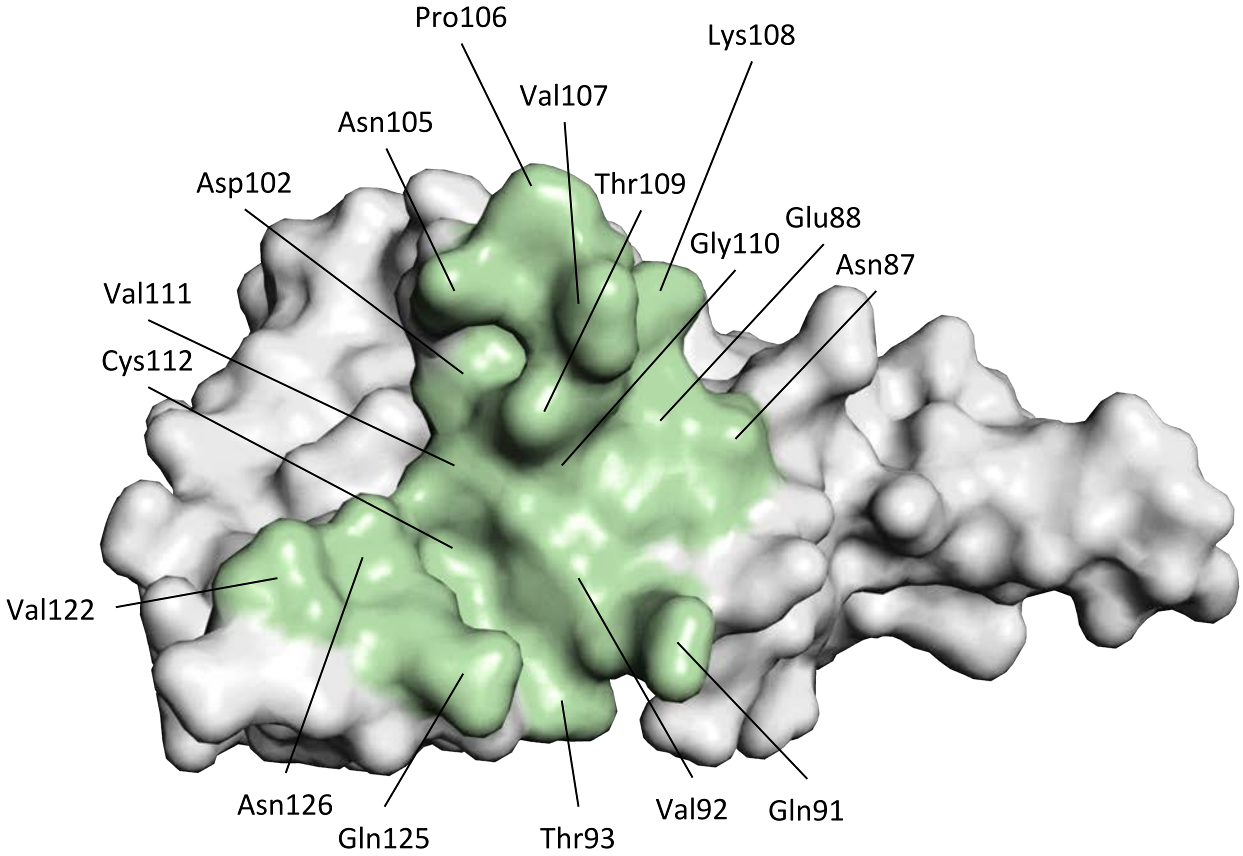

Supplementary Figure 9. Pfs25 residues involved in recognition by site 1a mAbs. Pfs25 is shown as a surface representation and EGF-like domain 3 residues that are contacted by 1190, 1262, 1269 and 1276 are colored green and their position labeled.

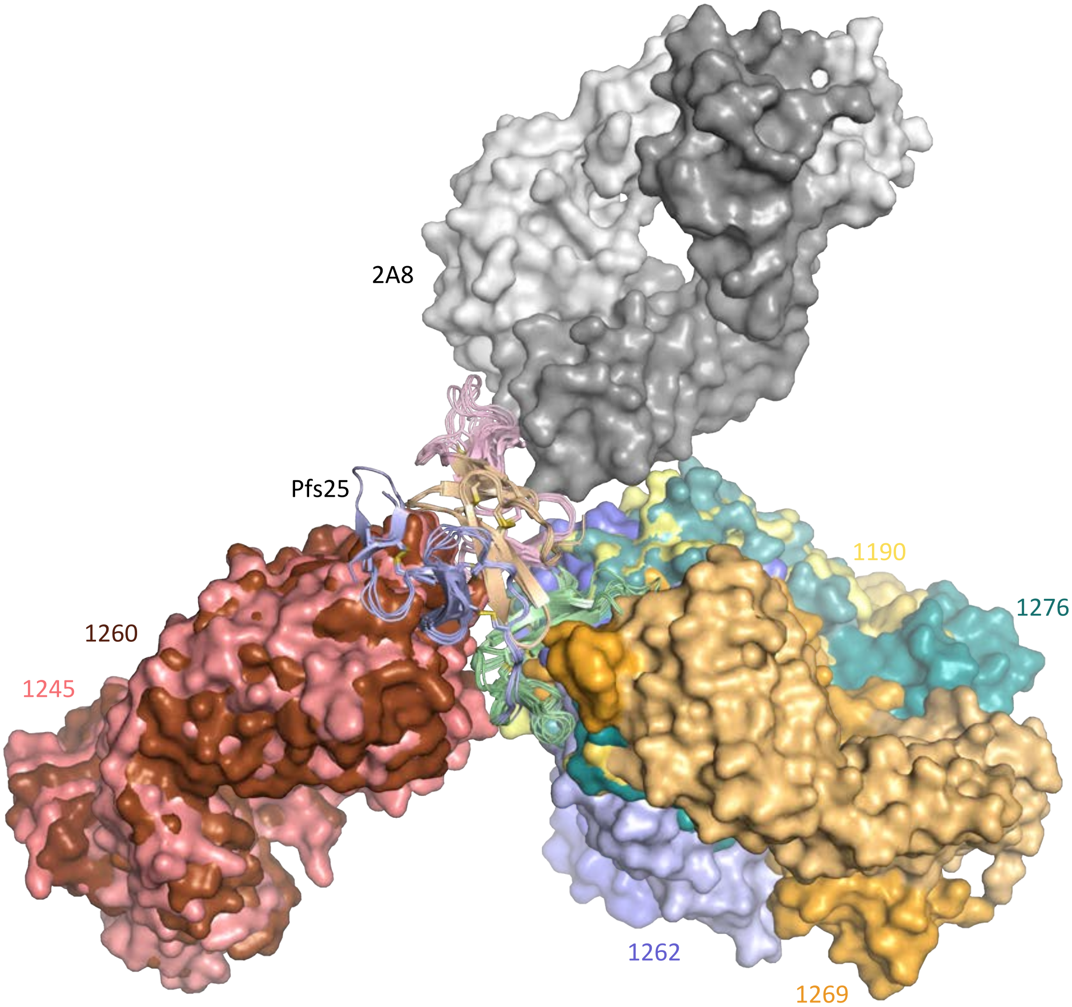

Supplementary Figure 10. Superposition of Pfs25-Fab co-complex crystal structures with the Pvs25-2A8 crystal structure. Pfs25-Fab crystal structures have been superposed with the Pvs25-2A8 crystal structure (PDB ID 1Z3G)[3]. Fabs are shown as surface representation and are colored as in Fig. 3. 2A8 is colored in grey and is shown to bind Pvs25 to a site distal to site 1 and site 2 on Pfs25.

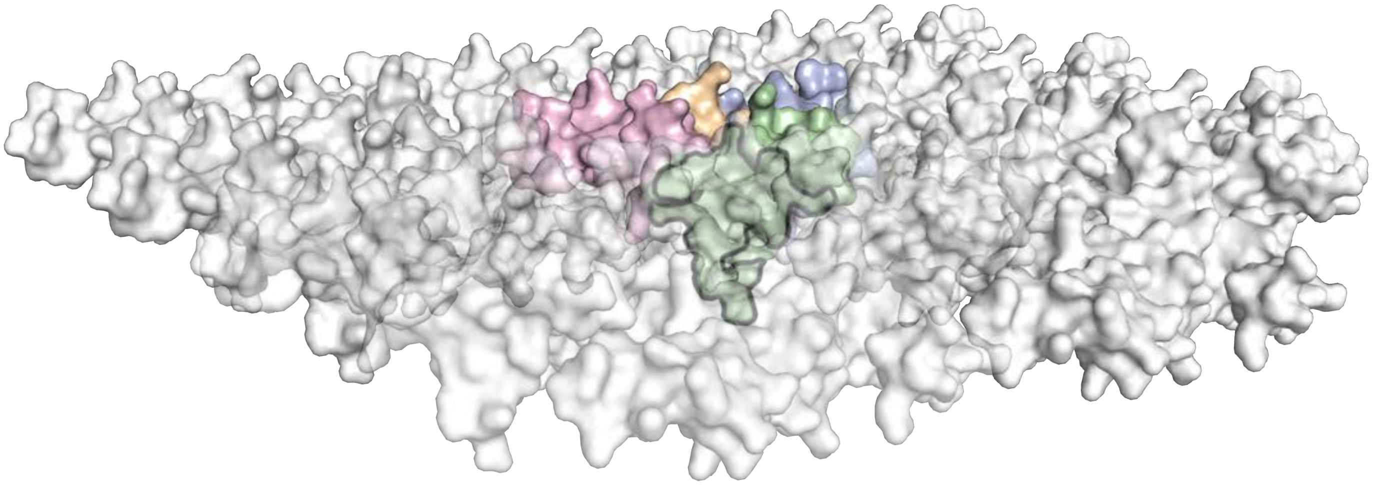

Supplementary Figure 11. The proposed Pvs25 tile-like packing model is incompatible with Pfs25 recognition by Kymice-derived, site 1-directed mAbs. The tile-like crystal packing present in the Pvs25 crystal structure (PDB 1Z1Y)[3]. The central Pvs25 molecule is colored according to EGF-like domain (wheat – EGF-like domain 1, pink – EGF-like domain 2, green – EGF-like domain 3, blue – EGF-like domain 4), while surrounding Pvs25 molecules are colored grey and are transparent to aid visibility. The interaction area of site 1a antibodies is outlined in black. While site 2 antibodies could bind to the outward facing triangular face of the proposed Ps25 tile-like arrangement, the epitopes of site 1-directed antibodies are inaccessible, making this suggested disposition of Ps25 on the surface of ookinetes unlikely for Pfs25.

Supplementary Table 1: SMFA of mAbs tested at 375 and 10 μg/mL

Test mAb	mAb conc. [μg/mL]	Mean oocysts	% Inhibition (%TRA)[a]			
			Best-estimate	95%CI Lo	95%CI Hi	p-value
Assay #1						
Control	N/A	23.5				
973	128	4.8	79.6	42.2	93.3	0.006
981	375	21.6	8.3	-148.8	67.6	0.867
1185	375	23.4	0.6	-204.5	66.7	0.967
1189	375	19.6	16.8	-152.2	72.5	0.757
1190	375	10.8	54.3	-31.6	84.0	0.168
1192	375	20.6	12.6	-152.5	68.3	0.786
1195	375	17.3	26.4	-109.0	74.6	0.600
1198	375	14.5	38.5	-75.8	79.3	0.368
1199	375	21.4	8.9	-160.7	68.6	0.818
1202	375	0.3	98.7	95.9	99.8	0.001
1203	375	23.2	1.3	-185.5	68.0	0.985
1207	375	21.8	7.4	-175.8	67.8	0.814
1223	375	21.4	8.9	-167.5	66.5	0.865
1224	375	19.3	18.1	-139.0	72.6	0.706
1226	375	29.2	-24.3	-247.6	59.5	0.661
1230	375	16.3	30.6	-106.6	75.3	0.493
1231	375	17.6	25.3	-119.1	76.2	0.587
1243	375	16.8	28.7	-94.8	77.1	0.538
1244	375	0.0	100.0	99.3	100.0	0.001
1245	375	1.6	93.2	79.5	98.2	0.001
Assay #2						
Control	N/A	6.5				
1260	375	0.1	99.2	97.0	100.0	0.001
1261	375	0.2	97.7	89.7	100.0	0.001
1262	375	0.0	100.0	97.6	100.0	0.001
1263	375	0.1	99.2	95.5	100.0	0.001
1264	375	0.1	98.4	92.0	100.0	0.001
1265	375	0.1	99.2	94.7	100.0	0.001
1266	375	0.1	99.2	94.7	100.0	0.001
1267	375	0.2	97.7	91.0	100.0	0.001
1268	375	0.5	93.0	79.7	98.0	0.001
1269	375	0.0	100.0	98.1	100.0	0.001
1270	375	0.2	97.7	92.7	99.4	0.001
1271	375	0.1	98.4	94.1	100.0	0.001
1272	375	0.1	99.2	97.3	100.0	0.001
1273	375	0.1	98.4	94.4	100.0	0.001
1276	375	0.2	97.7	91.6	99.7	0.001

Assay #3						
Control	N/A	45.0				
1202	10	70.2	-56.1	-342.5	44.9	0.405
1244	10	42.5	5.5	-175.1	67.0	0.957
1245	10	62.7	-39.5	-308.8	52.3	0.528
1260	10	67.5	-50.2	-349.2	50.6	0.470
1261	10	84.3	-87.5	-432.5	34.8	0.241
1262	10	56.3	-25.1	-270.4	55.5	0.668
1263	10	75.5	-67.9	-367.8	41.8	0.351
1264	10	58.1	-29.3	-257.9	56.3	0.643
1265	10	56.9	-26.5	-250.4	57.0	0.686
1266	10	73.1	-62.5	-383.8	41.2	0.355
1267	10	37.2	17.4	-146.5	73.0	0.752
1268	10	62.9	-39.8	-321.2	52.1	0.538
1269	10	55.9	-24.4	-252.5	57.3	0.688
1270	10	33.9	24.7	-107.3	72.3	0.593
1271	10	57.3	-27.5	-280.8	57.4	0.670
1272	10	74.1	-64.8	-387.0	42.5	0.358
1273	10	56.2	-25.0	-256.7	58.2	0.667
1276	10	54.3	-20.8	-252.8	57.1	0.720

[a] The best estimate and 95%CI of % inhibition, and the p-value (whether the observed inhibition was significantly different from zero) were calculated using a zero-inflated negative binomial model[5].

[b] Since AB1190 did not show a strong inhibition in Assay #1, the mAb was not tested at 10 µg/mL.

Supplementary Table 2: Binding kinetics of Fabs to Pfs25.

Fabs	K_D (nM)	k_a (1/Ms)	k_d (1/s)
**1190**	6.7 ± 0.7	$3.0\times10^{5} \pm 1.8\times10^{4}$	$2.1\times10^{-3} \pm 3.0\times10^{-4}$
**1202**	35.0 ± 7.0	$4.8\times10^{5} \pm 2.6\times10^{4}$	$1.6\times10^{-2} \pm 2.7\times10^{-3}$
**1245**	31.0 ± 5.6	$2.2\times10^{4} \pm 1.7\times10^{3}$	$3.8\times10^{-4} \pm 1.4\times10^{-4}$
**1260**	41.0 ± 9.8	$2.3\times10^{4} \pm 1.3\times10^{3}$	$9.3\times10^{-4} \pm 2.0\times10^{-4}$
**1262**	12.0 ± 0.4	$8.0\times10^{4} \pm 1.3\times10^{4}$	$1.1\times10^{-3} \pm 9.0\times10^{-5}$
**1266**	31.0 ± 7.0	$4.2\times10^{5} \pm 8.3\times10^{4}$	$1.2\times10^{-2} \pm 9.6\times10^{-4}$
**1267**	7.9 ± 1.2	$4.3\times10^{5} \pm 8.4\times10^{4}$	$3.2\times10^{-3} \pm 4.0\times10^{-5}$
**1268**	67 ± 15	$9.3\times10^{4} \pm 3.4\times10^{3}$	$6.0\times10^{-3} \pm 1.2\times10^{-3}$
**1269**	3.7 ± 0.3	$2.2\times10^{5} \pm 2.7\times10^{4}$	$8.4\times10^{-4} \pm 1.6\times10^{-4}$
**1276**	16.0 ± 2.5	$2.1\times10^{5} \pm 3.1\times10^{4}$	$3.7\times10^{-3} \pm 9.6\times10^{-4}$

Supplementary Table 3: Data collection and refinement statistics.

	Pfs25-1190	Pfs25-1245	Pfs25-1260	Pfs25-1262	Pfs25-1269	Pfs25-1276
**Wavelength (Å)**	0.97949	0.97949	0.97949	0.97949	0.97949	0.97949
**Space group**	$P2_1$	$P2_1$	$P2_1$	$P2_12_12_1$	$P2_1$	C2
**Cell dimensions**						
***a,b,c* (Å)**	53.6 203.1 65.5	51.8 83.7 76.2	53.2 79.6 78.1	65.1 119.0 165.0	82.1 39.5 101.8	139.4 70.3 83.7
**α, β, γ (°)**	90 109.9 90	90 93.7 90	90 90.9 90	90 90 90	90 94.7 90	90 90.5 90
**Resolution (Å)[a]**	40-2.4 (2.5-2.4)	40-1.9 (2.0-1.9)	40-3.3 (3.4-3.3)	47-2.7 (2.8-2.7)	40-2.5 (2.6-2.5)	40-2.2 (2.3-2.2)
**No. molecules in ASU**	2	1	1	2	1	1
**No. unique observations**	51,230 (5,894)	51,093 (7,230)	9,926 (835)	36,020 (3,525)	23,042 (2,539)	40,434 (5,096)
**Multiplicity**	3.9 (3.9)	3.8 (3.8)	3.8 (3.8)	7.4 (7.5)	3.7 (3.8)	3.1 (3.1)
**R_{merge} (%)[b]**	11.6 (55.8)	11.0 (58.7)	7.3 (60.9)	22.6 (110.8)	5.0 (48.6)	4.8 (42.1)
**R_{pim} (%)[c]**	6.8 (33.0)	6.5 (35.1)	4.4 (36.2)	8.9 (43.4)	3.0 (29.4)	3.3 (30.0)
**<I/σ I>**	11.5 (2.0)	11.8 (2.2)	14.6 (1.5)	9.7 (2.1)	17.4 (2.1)	16.3 (2.0)
**CC½**	99.4 (64.4)	99.6 (61.0)	99.8 (66.4)	98.8 (66.9)	99.9 (88.7)	99.8 (87.0)
**Completeness (%)**	99.9 (99.9)	99.8 (99.8)	99.8 (100)	99.9 (100)	99.6 (99.9)	98.0 (99.4)
**Refinement Statistics**						
**Non-hydrogen atoms**	9,210	5,121	4,204	9,388	4,393	4,669
**Macromolecule**	8,740	4,545	4,204	8,933	4,366	4,476
**Water**	416	546	-	443	15	169
**Ligand**	54	30	-	12	12	24
**R_{factor}[d] / R_{free}[e]**	19.1 / 21.7	16.4 / 20.0	23.0 / 27.9	18.6 / 23.8	21.7 / 24.9	19.1 / 23.3
**Rms deviations from ideality**						
**Bond lengths (Å)**	0.002	0.011	0.002	0.004	0.004	0.005
**Bond angle (°)**	0.55	1.11	0.49	0.65	0.96	0.71
**Ramachandran plot**						
**Favoured regions (%)**	97.4	98.1	95.1	97.3	94.8	96.3
**Allowed regions (%)**	2.6	1.9	4.9	2.7	5.2	3.7
**B-factors ($Å^2$)**						
**Average B-factors**	46.3	28.1	136.8	39.2	80.5	64.3
**Average macromolecule**	46.5	26.9	136.8	39.5	80.5	64.6
**Average ligand**	55.4	52.1	-	58.6	100.0	82.7
**Average water molecule**	39.8	36.4	-	32.8	60.0	54.2

a Values in parentheses refer to the highest resolution bin.

b $R_{merge} = \Sigma_{hkl} \Sigma_i \mid I_{hkl,i} - \langle I_{hkl} \rangle \mid / \Sigma_{hkl} \langle I_{hkl} \rangle$

c $R_{pim} = \Sigma_{hkl} [1/(N-1)]^{1/2} \Sigma_i \mid I_{hkl,i} - \langle I_{hkl} \rangle \mid / \Sigma_{hkl} \langle I_{hkl} \rangle$

d $R_{factor} = (\Sigma \mid |F_o| - |F_c| \mid) / (\Sigma \mid |F_o|)$ - for all data except as indicated in footnote e.

e 5% of data were used for the R_{free} calculation.

Supplementary Table 4: Table of contacts between 1190 and Pfs25 (K_D = 6.7 ± 0.7 nM).

Pfs25 Residue (BSA $Å^2$)	Interaction Type	1190-Fab Residue
**Gly76 (13.13)**		
Gly	VDW	L-Tyr52
**Tyr77 (4.96)**		
Tyr	VDW	L-Tyr52
**Asn87 (75.85)**		
Asn	VDW	L-Ser32, L-Tyr49, L-Tyr50, L-Asp51, L-Tyr52, L-Asp53
Asn$^{N\delta2A}$	HB	L-Tyr50^{O}
Asn$^{N\delta2B}$	HB	L-Tyr50^{O}, L-Asp51$^{O\delta2}$
**Glu88 (39.79)**		
Glu	VDW	L-Tyr50, L-Asp53
**Lys90 (104.02)**		
Lys	VDW	L-Ile28, L-Gly29, L-Ser30, L-Lys31, L-Ser32, L-Asp51, L-Asn66
Lys$^{N\zeta}$	SB	L-Asp$^{O\delta1}$, L-Asp$^{O\delta2}$
Lys$^{N\zeta}$	HB	L-Gly29^{O}, L-Lys30^{O}
**Gln91 (171.09)**		
Gln	VDW	L-Ser30, L-Lys31, L-Ser32, L-Trp91, L-Asp92, L-Ser93, H-Thr99, H-Ala100
Gln$^{O\varepsilon1}$	HB	L-Ser32^{N}
Gln$^{O\varepsilon1}$	WMHB	L-Ser32^{O}, L-Trp91^{N}
Gln$^{N\varepsilon2}$	HB	L-Trp91^{O}, L-Ser32$^{O\gamma}$
**Val92 (33.39)**		
Val	VDW	H-Thr98, H-Thr99
**Thr93 (2.57)**		
Thr	VDW	H-Thr99
**Asp102 (19.63)**		
Asp	VDW	H-Arg96
Asp$^{O\delta2}$	SB	H-Arg96$^{N\varepsilon}$
**Asn105 (55.21)**		
Asn	VDW	H-Tyr32, H-Arg94, H-Asp101
Asn$^{O\delta1}$	HB	H-Arg94$^{N\eta2}$
Asn$^{N\delta2}$	HB	H-Tyr32^{OH}
**Pro106 (40.23)**		
Pro	VDW	L-Tyr49, L-Ser56, H-Tyr102
**Val107 (118.15)**		
Val	VDW	L-Leu46, L-Tyr49, L-Pro55, H-Pro100B, H-Asp101
**Lys108 (45.33)**		
Lys	VDW	L-Tyr49, L-Tyr50, L-Asp53, H-Pro100B
Lys^{N}	HB	L-Tyr49^{OH}
**Thr109 (64.78)**		

Thr	VDW	L-Tyr50, H-Arg96, H-Thr98, H-Ala100A, H-Pro100B
**Gly110 (17.84)**		
Gly	VDW	L-Tyr50, H-Thr98, H-Thr99
Gly^{N}	HB	$L\text{-}Tyr50^{OH}$
Gly^{O}	HB	$H\text{-}Thr98^{O\gamma1}$
Gly^{O}	WMHB	$L\text{-}Tyr50^{OH}$
**Val111 (36.32)**		
Val	VDW	H-Arg96, H-Ile97, H-Thr98
**Cys112 (1.27)**		
Cys	VDW	H-Thr98
**Val122 (85.00)**		
Val	VDW	H-Thr30, H-Tyr32, H-Asn52, H-Asn53, H-Ile97
Val^{O}	HB	$H\text{-}Asn52^{N\delta2}$
**Gln123 (52.45)**		
Gln	VDW	H-Asn52, H-Thr53, H-Asp54
$Gln^{N\varepsilon2}$	HB	$H\text{-}Asp54^{O\delta1}$
**Gln125 (149.32)**		
Gln	VDW	L-Trp91, L-His95B, H-Tyr33, H-Trp50, H-Ile97, H-Thr98, H-Thr99, H-Ala100
$Gln^{N\varepsilon2}$	HB	$H\text{-}Thr98^{O}$
**Asn126 (51.34)**		
Asn	VDW	H-Arg96, H-Ile97, H-Thr98
$Asn^{N\delta2}$	HB	$H\text{-}Ile97^{O}$
$Asn^{N\delta2}$	WMHB	$H\text{-}Asp31^{O\delta2}$
**Lys127 (9.72)**		
Lys	VDW	H-Thr99

Supplementary Table 5: Table of contacts between 1276 and Pfs25 (K_D = 16.0 ± 2.5 nM).

Pfs25 Residue (BSA Å^2)	Interaction Type	1276-Fab Residue
**Leu75 (11.52)**		
Leu	VDW	L-Phe52
**Gly76 (11.75)**		
Gly	VDW	L-Phe52
**Asp78 (10.92)**		
Asp	VDW	L-Asp51, L-Phe52
**Asn87 (65.57)**		
Asn	VDW	L-Ser32, L-Tyr50, L-Asp51, L-Phe52, L-Asp53
Asn$^{N\delta2}$	HB	L-Tyr50^{O}
Asn$^{N\delta2}$	HB	L-Asp53$^{O\delta1}$
**Glu88 (41.74)**		
Glu	VDW	L-Ser32, L-Tyr50, L-Asp53
**Lys90 (95.17)**		
Lys	VDW	L-Gly29, L-Ser30, L-Lys31, L-Ser32, L-Asp51, L-Asn66
Lys$^{N\zeta}$	SB	L-Asp$^{O\delta1}$, L-Asp$^{O\delta2}$
Lys$^{N\zeta}$	HB	L-Gly29^{O}
**Gln91 (167.22)**		
Gln	VDW	L-Ser30, L-Lys31, L-Ser32, L-Trp91, L-Asp92, L-Ser93, H-Thr99, H-Ala100
Gln$^{O\varepsilon1}$	HB	L-Ser32^{N}
Gln$^{O\varepsilon1}$	WMHB	L-Ser32^{O}, L-Trp91^{N}
Gln$^{N\varepsilon2}$	HB	L-Trp91^{O}
**Val92 (37.47)**		
Val	VDW	H-Thr98, H-Thr99
**Thr93 (2.34)**		
Thr	VDW	H-Thr99
**Asp102 (8.76)**		
Asp	VDW	H-Arg96
**Asn105 (50.17)**		
Asn	VDW	H-Tyr32, H-Arg94, H-Asp101
Asn$^{O\delta1}$	HB	H-Arg94$^{N\eta2}$
Asn$^{N\delta2}$	HB	H-Tyr32^{OH}
**Pro106 (31.85)**		
Pro	VDW	L-Tyr49
Pro^{O}	HB	L-Tyr49^{OH}
**Val107 (118.44)**		
Val	VDW	L-Leu46, L-Tyr49, L-Pro55, H-Pro100B, H-Asp101
**Lys108 (42.81)**		
Lys	VDW	L-Tyr49, L-Tyr50, L-Asp53, H-Pro100B

Lys^{N}	HB	L-$Tyr49^{OH}$
**Thr109 (64.86)**		
Thr	VDW	L-Tyr50, H-Arg96, H-Thr98, H-Ala100A, H-Pro100B
**Gly110 (22.3)**		
Gly	VDW	L-Tyr50, H-Thr98, H-Thr99
Gly^{N}	HB	L-$Tyr50^{OH}$
Gly^{O}	HB	H-$Thr98^{O\gamma1}$
Gly^{O}	WMHB	L-$Tyr50^{OH}$
**Val111 (32.44)**		
Val	VDW	H-Arg96, H-Thr98,
**Cys112 (2.55)**		
Cys	VDW	H-Thr98
**Val122 (81.63)**		
Val	VDW	H-Thr30, H-Tyr32, H-Asn52, H-Asn53, H-Ile97
Val^{O}	HB	H-$Asn52^{N\delta2}$
**Gln123 (43.02)**		
Gln	VDW	H-Asn52, H-Ser54
**Gln125 (149.07)**		
Gln	VDW	L-Trp91, L-Arg95, H-Tyr33, H-Trp50, H-Ile97, H-Thr98, H-Thr99, H-Ala100
$Gln^{O\varepsilon1}$	HB	H-$Thr99^{O\gamma1}$
**Asn126 (47.77)**		
Asn	VDW	H-Arg96, H-Ile97, H-Thr98
$Asn^{N\delta2}$	HB	H-$Ile97^{O}$
**Lys127 (11.38)**		
Lys	VDW	H-Thr99

Supplementary Table 6: Table of contacts between 1262 and Pfs25 (K_D = 12.0 ± 0.4 nM).

Pfs25 Residue (BSA $Å^2$)	Interaction Type	1262-Fab Residue
**Asn87 (57.45)**		
Asn	VDW	H-His33, H-Trp34
**Glu88 (42.21)**		
Glu	VDW	H-Trp34, H-Arg97, H-Phe98
**Lys90 (68.23)**		
Lys	VDW	H-Ser30, H-Ser31, H-Ser32, H-His33, H-Leu53
**Gln91 (63.88)**		
Gln	VDW	H-Ser31, H-Ser32, H-Arg94
$Gln^{N\varepsilon2}$	HB	$H\text{-}Ser31^{O}$
**Val92 (31.84)**		
Val	VDW	H-Phe98
**Thr93 (3.27)**		
Thr	VDW	H-Phe98
**Asp102 (11.30)**		
Asp	VDW	L-Tyr32
$Asp^{O\delta2}$	HB	$L\text{-}Tyr32^{OH}$
**Asn105 (64.13)**		
Asn	VDW	L-Ile29, L-Asn30, L-Tyr32, L-Asn92
$Asn^{O\delta1}$	HB	$L\text{-}Asn30^{N\delta2}$, $L\text{-}Asn92^{N\delta2}$
**Pro106 (34.57)**		
Pro	VDW	L-Asn92, L-Ser93
**Val107 (118.89)**		
Val	VDW	L-Leu91, L-Asn92, L-Ser93, L-Tyr94, H-Arg97, H-Ala100A
Val^{N}	HB	$L\text{-}Asn92^{O}$
**Lys108 (9.46)**		
Lys	VDW	H-Arg97
**Thr109 (59.51)**		
Thr	VDW	L-Tyr32, H-Arg97, H-Phe98 H-Tyr99, H-Gly100
$Thr^{O\gamma1}$	HB	$H\text{-}Arg97^{O}$, $H\text{-}Tyr99^{N}$, $H\text{-}Gly100^{N}$, $H\text{-}Gly100^{O}$
**Gly110 (23.54)**		
Gly	VDW	H-Arg97, H-Phe98, H-Tyr99
Gly^{N}	HB	$H\text{-}Arg97^{O}$
Gly^{O}	HB	$H\text{-}Tyr99^{N}$
**Val111 (46.10)**		
Val	VDW	L-Tyr32, H-Phe98, H-Tyr99
**Cys112 (11.13)**		
Cys	VDW	H-Phe98, H-Tyr99
Cys^{N}	HB	$H\text{-}Tyr99^{OH}$

Cys^{O}	HB	H-Tyr99^{OH}
**Pro120 (9.37)**		
Pro	VDW	H-Tyr99
**Val122 (62.27)**		
Val	VDW	L-Ser52, L-Thr53, L-Leu54
**Gln125 (106.13)**		
Gln	VDW	L-Tyr49, L-Thr53, L-Leu54, L-Gln55, L-Ser56, H-Phe98
$Gln^{N\varepsilon 2}$	HB	L-Leu54^{O}
**Asn126 (79.93)**		
Asn	VDW	L-Tyr49, L-Ala50, L-Thr53, H-Phe98, H-Tyr99
$Asn^{O\delta 1}$	HB	L-Thr53$^{O\gamma 1}$

Supplementary Table 7: Table of contacts between 1269 and Pfs25 (K_D = 3.7 ± 0.3 nM).

Pfs25 Residue (BSA Å^2)	Interaction Type	1269-Fab Residue
**Ser18 (16.62)**		
Ser	VDW	H-Phe99, H-Tyr100D
**Gly19 (35.43)**		
Gly	VDW	H-Tyr100D
**Asn87 (43.94)**		
Asn	VDW	H-Ser53, H-Asp55
Asn$^{N\delta2}$	HB	H-Ser53$^{O\gamma}$, H-Asp55$^{O\delta2}$
**Glu88 (58.73)**		
Glu	VDW	H-Ser52, H-Gly52A, H-Ser53, H-Gly54, H-Asp55, H-Ser56
Glu$^{O\varepsilon1}$	HB	H-Gly52A^{N}, H-Ser53^{N}
Glu$^{O\varepsilon2}$	HB	H-Ser52$^{O\gamma1}$, H-Ser53$^{O\gamma1,}$ H-Ser56$^{O\gamma1}$
**Gln91 (90.94)**		
Gln	VDW	H-Thr28, H-Ser30, H-Arg31, H-Gly52A, H-Ser53, H-Asn73
**Val92 (34.13)**		
Val	VDW	H-Arg31
**Thr93 (9.51)**		
Thr	VDW	H-Arg31
Thr^{O}	HB	H-Arg31$^{N\eta2}$
**Ile100 (3.68)**		
Ile	VDW	H-Tyr100D
**Asp102 (32.89)**		
Asp	VDW	L-Tyr32, H-Tyr100D, H-Phe100F
Asp$^{O\delta1}$	HB	L-Tyr32^{OH}
**Ser104 (26.34)**		
Ser	VDW	L-Tyr32
**Asn105 (52.06)**		
Asn	VDW	L-Tyr32, L-Tyr91, H-Phe100F
**Pro106 (83.01)**		
Pro	VDW	L-Tyr32, L-Gln90, L-Tyr91, H-Tyr58
**Val107 (101.94)**		
Val	VDW	L-Tyr91, L-Gly92, H-Ala33, H-Thr50, H-Tyr58
**Lys108 (47.52)**		
Lys	VDW	H-Ser52, H-Gly52A, H-Ser56, H-Tyr58
Lys^{N}	HB	H-Tyr58^{OH}
**Thr109 (41.45)**		
Thr	VDW	L-Tyr91, H-Phe100F
**Gly110 (11.21)**		
Gly	VDW	H-Arg31, H-Tyr97
**Val111 (53.68)**		

Val	VDW	H-Tyr97, H-Tyr100D, H-Phe100F
**Cys112 (12.76)**		
Cys	VDW	H-Arg31, H-Tyr97, H-Tyr100D
Cys^{N}	HB	H-$Tyr97^{OH}$
Cys^{O}	HB	H-$Tyr100D^{OH}$
**Ser113 (0.69)**		
Ser	VDW	H-Tyr100D
**Val119 (18.47)**		
Val	VDW	H-Phe99
**Pro120 (50.04)**		
Pro	VDW	H-Tyr97, H-Phe99, H-Tyr100D
**Val122 (76.54)**		
Val	VDW	L-Tyr49, L-Thr56, H-Tyr97, H-Tyr98,
**Gln123 (128.84)**		
Gln	VDW	L-Thr56
$Gln^{N\varepsilon 2}$	HB	L-$Thr56^{O\gamma 1}$
**Gln125 (99.08)**		
Gln	VDW	H-Phe27, H-Thr28, H-Arg31, H-Asn32
Gln^{O}	HB	H-$Arg31^{N\eta 2}$
$Gln^{O\varepsilon 1}$	HB	H-$Thr28^{N}$
$Gln^{N\varepsilon 2}$	HB	H-$Thr28^{O\gamma 1}$
**Asn126 (72.93)**		
Asn	VDW	H-Arg31, H-Asp96, H-Tyr97, H-Tyr98
Asn^{O}	HB	H-$Tyr97^{OH}$
$Asn^{N\delta 2}$	HB	H-$Asp96^{O\delta 2}$, H-$Tyr97^{O}$
**Lys127 (7.60)**		
Lys	VDW	H-Arg31

Supplementary Table 8: Table of contacts between 1245 and Pfs25 (K_D = 31.0 ± 5.6 nM).

Pfs25 Residue (BSA Å²)	Interaction Type	1245-Fab Residue
**Phe13 (31.08)**		
Phe	VDW	H-Tyr100A, H-Tyr100C
**Ile15 (12.72)**		
Ile	VDW	H-Tyr100C, H-Tyr100D
**Met17 (10.48)**		
Met	VDW	H-Tyr100D
**Glu22 (12.87)**		
Glu	VDW	H-Tyr100D
$Glu^{o\varepsilon2}$	HB	$H\text{-}Tyr100D^{OH}$
**Lys24 (56.33)**		
Lys	VDW	H-Tyr100C, H-Arg100B, H-Tyr100A
$Lys^{N\zeta}$	HB	$H\text{-}Arg100B^{O}$
$Lys^{N\zeta}$	WMHB	$L\text{-}Asp28^{O\delta2}$
**Cys25 (4.48)**		
Cys	VDW	H-Tyr100A
**Glu26 (7.28)**		
Glu	VDW	H-Tyr100A
**Asn27 (21.20)**		
Asn	VDW	H-Tyr100A
Asn^{N}	HB	$H\text{-}Tyr100A^{OH}$
**Asp28 (16.73)**		
Asp	VDW	L-Ser27E
**Lys40 (33.97)**		
Lys	VDW	L-Ser27E
$Lys^{N\zeta}$	HB	$L\text{- }Ser27E^{O}$
**Asn81 (7.45)**		
Asn	VDW	L-Gly29
Asn^{O}	WMHB	$L\text{-}Asn30^{O\delta1}$
**Asn82 (48.42)**		
Asn	VDW	L-Gly29, L-Asp28, L-Val27C, L-Thr31
$Asn^{O\delta1}$	WMHB	$L\text{-}Thr31^{O\gamma1}$
**Val83 (40.58)**		
Val	VDW	L-Asn30, L-Asp28, L-Gly29
**Gly95 (23.4)**		
Gly	VDW	L-Tyr49
Gly^{O}	HB	$L\text{-}Tyr49^{OH}$
Gly^{O}	WMHB	$H\text{-}Tyr100D^{O}$
**Asn96 (6.93)**		
Asn	VDW	L-Tyr49
**Gly97 (12.02)**		
Gly	VDW	H-Tyr100D
Gly^{O}	WMHB	$H\text{-}Tyr100D^{O}$

**Lys98 (29.29)**		
Lys	VDW	H-Tyr100D
$Lys^{N\zeta}$	HB	$H\text{-}Tyr100D^{OH}$
**Asn115 (38.10)**		
Asn	VDW	H-Tyr100D, H-Tyr100E
$Asn^{N\delta2}$	HB	$H\text{-}Tyr100D^{O}$
$Asn^{O\delta1}$	WMHB	$H\text{-}Tyr100D^{N}$
**Ile116 (5.26)**		
Ile	VDW	H-Tyr32
**Lys148 (17.60)**		
Lys	VDW	H-Tyr53
**Ala149 (35.84)**		
Ala	VDW	H-Tyr53, H-Ser31, H-Thr30
Ala^{O}	HB	$H\text{-}Ser31^{O\gamma}$
**Val150 (66.47)**		
Val	VDW	H-Tyr53, H-Ser31, H-Tyr100C, H-Asp98, H-Gly97
**Asp151 (134.57)**		
Asp	VDW	H-Ser31, H-Tyr32, H-Tyr100C, H-Tyr100E, H-Tyr100D, H-Asp98, H-Gly97, H-Arg96
Asp^{N}	HB	$H\text{-}Ser31^{O}$
$Asp^{O\delta1}$	HB	$H\text{-}Gly97^{N}$
$Asp^{O\delta2}$	HB	$H\text{-}Asp98^{N}$
$Asp^{O\delta2}$	WMHB	$H\text{-}Arg96^{N\varepsilon}$, $H\text{-}Tyr100A^{O}$
**Gly152 (15.96)**		
Gly	VDW	H-Tyr32, H-Tyr100E
Gly^{N}	HB	$H\text{-}Tyr32^{OH}$
**Ile153 (26.44)**		
Ile	VDW	H-Tyr100C, H-Tyr100D
**Lys155 (31.97)**		
Lys	VDW	H-Tyr53, H-Tyr100C, H-Tyr100A, H-Asp98
$Lys^{N\zeta}$	HB	$H\text{-}Tyr100C^{OH}$
$Lys^{N\zeta}$	SB	$H\text{-}Asp98^{O\delta2}$
**Asp157 (17.90)**		
Asp	VDW	H-Tyr53
$Asp^{O\delta2}$	HB	$H\text{-}Tyr53^{OH}$

Supplementary Table 9: Table of contacts between 1260 and Pfs25 (K_D = 41.0 ± 9.8 nM).

Pfs25 Residue (BSA Å^2)	Interaction Type	1260-Fab Residue
**Phe13 (32.28)**		
Phe	VDW	H-Tyr100A, H-Tyr100C
**Ile15 (15.57)**		
Ile	VDW	H-Tyr100C, H-Tyr100D
**Met17 (11.27)**		
Met	VDW	H-Tyr100D
**Glu22 (14.10)**		
Glu	VDW	H-Tyr100D
Glu$^{O\varepsilon2}$	HB	H-Tyr100D^{OH}
**Lys24 (57.48)**		
Lys	VDW	H-Tyr100C, H-Tyr100B
Lys$^{N\zeta}$	HB	H-Tyr100B^{O}
**Cys25 (4.82)**		
Cys	VDW	H-Tyr100A
**Glu26 (11.59)**		
Glu	VDW	H-Tyr100A
**Asn27 (16.77)**		
Asn	VDW	H-Tyr100A
**Asp28 (25.39)**		
Asp	VDW	L-Ser27E
**Asn81 (18.35)**		
Asn	VDW	L-Gly29, L-Ser52
**Asn82 (39.24)**		
Asn	VDW	L-Gly29
**Val83 (43.40)**		
Val	VDW	L-Asn30, L-Asp28, L-Gly29
**Gly95 (21.60)**		
Gly	VDW	L-Tyr49
Gly^{O}	HB	L-Tyr49^{OH}
**Gly97 (11.61)**		
Gly	VDW	H-Tyr100D
**Lys98 (30.24)**		
Lys	VDW	H-Tyr100D
Lys$^{N\zeta}$	HB	H-Tyr100D^{OH}
**Asn115 (39.67)**		
Asn	VDW	H-Tyr100D, H-Tyr100E
Asn$^{N\delta2}$	HB	H-Tyr100D^{O}
**Ile116 (5.75)**		
Ile	VDW	H-Tyr32
**Lys148 (32.42)**		
Lys	VDW	H-Tyr53, H-Asn54
**Ala149 (39.58)**		

Ala	VDW	H-Tyr53, H-Ser31, H-Thr30
Ala^{O}	HB	$H\text{-}Ser31^{O\gamma}$
**Val150 (65.19)**		
Val	VDW	H-Tyr53, H-Ser31, H-Tyr100C, H-Asp98, H-Gly97
**Asp151 (133.27)**		
Asp	VDW	H-Ser31, H-Tyr32, H-Tyr100C, H-Tyr100E, H-Tyr100D, H-Asp98, H-Gly97, H-Arg96, H-Asp95, H-Tyr100A
Asp^{N}	HB	$H\text{-}Ser31^{O}$
$Asp^{O\delta1}$	HB	$H\text{-}Gly97^{N}$
$Asp^{O\delta2}$	HB	$H\text{-}Asp98^{N}$
**Gly152 (16.85)**		
Gly	VDW	H-Tyr32, H-Tyr100E, H-Ser31
Gly^{N}	HB	$H\text{-}Tyr32^{OH}$
**Ile153 (25.60)**		
Ile	VDW	H-Tyr100C, H-Tyr100D
**Lys155 (33.99)**		
Lys	VDW	H-Tyr53, H-Tyr100C, H-Tyr100A, H-Asp98
$Lys^{N\zeta}$	HB	$H\text{-}Tyr100C^{OH}$
$Lys^{N\zeta}$	SB	$H\text{-}Asp98^{O\delta2}$
**Asp157 (22.82)**		
Asp	VDW	H-Tyr53, H-Asn54
$Asp^{O\delta2}$	HB	$H\text{-}Tyr53^{OH}$
**Ile163 (29.79)**		
Ile	VDW	H-Asn54

Supplementary Table 10: BSA (Å^2) and contact summary for Fab-Pfs25 crystal structures.

	H-bonds / Salt Bridge			BSA (Å^2)			
**Antibody**	**H-Chain**	**L-Chain**	**Total**	**H-Chain**	**L-Chain**	**Total**	**K_D**
**1269**	17 / 0	2 / 0	19 / 0	889.5	239.5	1129.0	3.7 ± 0.3
**1262**	9 / 0	6 / 0	15 / 0	534.8	394.8	929.6	12.0 ± 0.4
**1190**	7 / 1	10 / 2	17 / 3	621.7	515.1	1136.8	6.7 ± 0.7
**1276**	6 / 0	8 / 2	14 / 2	575.0	529.0	1104.0	16.0 ± 2.5
**1245**	12 / 1	2 / 0	14 / 1	614.4	188.7	803.1	31.0 ± 5.6
**1260**	11 / 1	1 / 0	12 / 1	681.6	166.0	847.6	41.0 ± 9.8

Supplementary Table 11: SMFA results of Feed #1 and #2.

Test mAb [µg/ml]		Mean oocysts	% Inhibition (%TRA)[a]			
AB1245	AB1269		Best-estimate	95%CI Lo	95%CI Hi	p-value
Feed #1						
0	0	36.7				
320	0	3.7	90.1	77.0	95.5	0.001
160	0	8.9	75.9	45.7	89.7	0.003
80	0	15.7	57.4	-1.2	81.1	0.051
40	0	18.1	50.8	-10.6	78.2	0.082
20	0	25.2	31.5	-57.3	71.0	0.357
10	0	30.7	16.3	-84.3	63.2	0.678
0	320	0.9	97.7	93.6	99.6	0.001
0	160	1.4	96.3	91.3	98.6	0.001
0	80	4.8	87.1	69.5	94.8	0.001
0	40	5.5	85.0	64.1	93.9	0.001
0	20	14.7	59.9	8.7	83.0	0.033
0	10	18.4	49.9	-12.5	77.6	0.094
Feed #2						
0	0	19.3				
350	0	3.9	80.1	54.2	91.3	0.001
140	0	6.2	67.9	26.6	86.2	0.004
56	0	17.5	9.3	-113.8	62.2	0.783
22	0	12.8	33.7	-46.6	70.9	0.320
9	0	20.5	-6.2	-142.5	55.4	0.876
0	350	0.5	97.4	93.9	99.0	0.001
0	140	1.2	94.0	86.4	97.5	0.001
0	56	3.9	80.1	53.3	91.3	0.002
0	22	8.9	53.9	-3.6	80.2	0.060
0	9	13.2	31.9	-55.7	69.9	0.335

[a] The best estimate and 95%CI of % inhibition, and the p-value (whether the observed inhibition was significantly different from zero) were calculated using a zero-inflated negative binomial model[5].

Supplementary Table 12: SMFA results of Feed #3 and #4.

Test mAb [µg/ml]		Mean oocysts	% Inhibition (%TRA)[a]			
AB1245	AB1269		Best-estimate	95%CI Lo	95%CI Hi	p-value
Feed #3						
0	0	20.2				
441	0	1.1	94.5	87.2	97.8	0.001
230	0	3.9	80.6	55.2	91.8	0.001
132	0	5.4	73.2	38.6	88.4	0.002
73	0	9.8	51.4	-14.0	78.4	0.078
45	0	10.9	45.9	-26.2	75.5	0.140
31	0	13.4	33.7	-47.5	73.3	0.325
0	110	2.5	87.6	71.4	94.7	0.001
0	53	2.5	87.8	72.8	94.9	0.001
0	27	8.8	56.6	-0.6	80.9	0.052
0	13	12.9	36.0	-42.9	70.2	0.284
0	7	12.7	37.0	-45.9	72.1	0.278
0	4	16.7	17.4	-86.7	63.8	0.698
132	27	2.0	90.3	76.3	96.1	0.001
73	13	4.0	80.4	54.4	91.4	0.001
45	7	10.2	49.4	-19.4	78.7	0.109
31	4	14.7	27.3	-63.7	68.5	0.437
Feed #4						
0	0	19.2				
441	0	3.9	79.6	53.9	91.3	0.003
230	0	7.7	59.8	8.0	82.3	0.032
132	0	12.6	34.5	-58.3	71.7	0.324
73	0	9.1	52.5	-10.5	81.5	0.088
45	0	14.0	27.2	-78.4	69.4	0.463
0	110	3.8	80.4	51.7	93.7	0.002
0	53	5.1	73.4	37.8	88.6	0.002
0	27	10.7	44.1	-32.7	75.9	0.165
0	13	14.6	24.0	-65.4	64.8	0.508
0	7	18.2	5.0	-114.3	57.5	0.915
132	27	3.3	82.9	54.2	94.0	0.002
73	13	7.0	63.4	18.7	83.9	0.010
45	7	8.8	53.9	-0.9	78.8	0.052

[a] The best estimate and 95%CI of % inhibition, and the p-value (whether the observed inhibition was significantly different from zero) were calculated using a zero-inflated negative binomial model[5].

Supplementary References

1. Sievers, F. *et al.* Fast, scalable generation of high-quality protein multiple sequence alignments using Clustal Omega. *Mol Syst Biol* **7,** 539–539 (2011).
2. Molecular Operating Environment (MOE), 2013.08; *Chemical Computing Group ULC,* 1010 Sherbooke St. West, Suite #910, Montreal, QC, Canada, H3A 2R7, 2017.
3. Saxena, A. K. *et al.* The essential mosquito-stage P25 and P28 proteins from Plasmodium form tile-like triangular prisms. *Nat. Struct. Mol. Biol.* **13,** 90–91 (2006).
4. Baker, N. A., Sept, D., Joseph, S., Holst, M. J. & McCammon, J. A. Electrostatics of nanosystems: Application to microtubules and the ribosome. *Proc. Natl. Acad. Sci. U.S.A.* **98,** 10037–10041 (2001).
5. Miura, K. *et al.* Transmission-blocking activity is determined by transmission-reducing activity and number of control oocysts in Plasmodium falciparum standard membrane-feeding assay. *Vaccine* **34,** 4145–4151 (2016).
