# Supplementary material for: Molecular definition of multiple sites of antibody inhibition of malaria transmission-blocking vaccine antigen Pfs25
Source: Nat Commun. 2017 Nov 16;8:1568. doi: 10.1038/s41467-017-01924-3 (PMC5691035; doi:10.1038/s41467-017-01924-3)
Supplement: Supplementary file 3 — Description of Additional Supplementary Files [file 41467_2017_1924_MOESM3_ESM.pdf]

File Name: Supplementary Data 1
Description: Anti-Pfs25 antibody variable sequences.
