# Supplementary material for: Molecular definition of multiple sites of antibody inhibition of malaria transmission-blocking vaccine antigen Pfs25
Source: Nat Commun. 2017 Nov 16;8:1568. doi: 10.1038/s41467-017-01924-3 (PMC5691035; doi:10.1038/s41467-017-01924-3)
Supplement: Supplementary file 4 — Supplementary Data 1 [file 41467_2017_1924_MOESM4_ESM.docx]

**Supplementary Data 1. Anti-Pfs25 antibody variable sequences.**

**>1190-Lambda-chain**

SYVLTQPPSVSVAPGQTARITCGGSNIGSKSVHWYQQKPGQTPMLVIYYDYDRPSGIPERFSGSNSGSTATLTISRVEAGDEADYYCQVWDSSSDHVWVFGGGTTLT

**>1190-Heavy-chain**

QVQLVQSGAEVKKPGASVKVSCKTSGYTFTDYYIHWVRQAPGQGLEWMGWINPTDGGTNYAQKFQDWVTMTRDTSITTAYMELSRLRSDGTAVYFCARDRITTAAPFDYWGQGTLVTVSS

**>1276-Lambda-chain**

SYVLTQPPSVSVAPGKTAKITCGGNNIGSKSVHWYQQKPGQAPVLVMYYDFDRPSGIPERFSGSNSGNTATLTISRVEAEDEADYYCQVWDSDRYWVFGGGTKLT

**>1276-Heavy-chain**

QVQLVQSGPEVKKPGASVKVSCKASGYTFTDYYIHWVRQAPGQGLEWMGWINPNSGGTNYAQNFQDWVTMTRDTSITTAYMELSSLRSDDTAVYYCARDRITTAAPFDYWGQGTLVTVSS

**>1245-Kappa-chain**

DVVMTQSPLSLPVTLGQPASISCRSSQSLVYSDGNTYLSWFQQRPGQSPRRLIYKVSNRDSGVPDRFSGSGSGTDFTLKISRVEAEDVGVYYCMQDTHWPPTFGGGTKVEIK

**>1245-Heavy-chain**

DVVMTQSPLSLPVTLGQPASISCRSSQSLVYSDGNTYLSWFQQRPGQSPRRLIYKVSNRDSGVPDRFSGSGSGTDFTLKISRVEAEDVGVYYCMQDTHWPPTFGGGTKVEIK

**>1260-Kappa-chain**

DVVMTQSPLSLPVTLGQPASISCRSSQSLVYSDGNTYLNWFQQGPGQSPRRLIYKVSNRDSGVPDRFSGSGSGTDFTLKISRVEAEDVGVYYCMQATHWPLTFGGGTKVEIK

**>1260-Heavy-chain**

QVQLVQSGAEVKKPGASVKVSCRASGYIFTSYGFSWVRQAPGQGLEWMGWISAYNGNTDYSQKLQGRVTMTTDTSTNTVYMELRTLQSDDTAVYYCARDRGDRLYYYYYYGMDVWGQGTTVTVSS

**>1266-Lambda-chain**

SYELTQPPSVSVSPGQSARITCSGDALPMKYAYWYQQKSGQAPVLVIYEDSKRPSGIPERFSGSSSGTMATLTISGAQVEDEADYYCFSTDSSGNHRVFGGGTKLT

**>1266-Heavy-chain**

QVQLQQSGPGLVKPSQTLSLTCAISGDSVSSNSAAWNWIRQSPSRGLEWLGRTYYRSKWYNDYAVSVKSRITINADTSKNQFSLQLNSVTPEDTAVYYCVRDLLGSSGWYVFFDNWGQGTLVTVSS

**>1267-Lambda-chain**

SYELTQPPSVSVSPGQSARITCSGDALPMKYAYWYQQKSGQAPVLVIYEDSKRPSGIPERFSGSSSGTMATLTISGAQVEDEADYYCFSTDSSGNHRVFGGGTKLT

**>1267-Heavy-chain**

QVQLQQSGPGLVKPSQTLSLTCAISGDSVSSNSAAWNWIRQSPSRGLEWLGRTYYRSKWYNDYAVSVRGRITINPDTSKNHFSLQLNSVTPEDTAVYFCARDLMTNSGWYVYFDCWGQGTLVTVSS

**>1202-Lambda-chain**

SYELTQPPSVSVSPGQTASITCSGDKLGDKYASWYQQKPGQSPVLVIFQDNKRPSGIPERFSGSNSGNTATLTISETQAMDEADYYCQAWDISTAVFGTGTNVT

**>1202-Heavy-chain**

QVQLVESGGDLVKPGGSLRLSCAASGFTFSDHYMNWIRQAPGKGLEWVSYISSSGSTIYYSDSVKGRFTISRDNAKYLLSLQMNSLRAEDTAVYFCARDPMVRGFHDAFDIWGQGTMVTVSS

**>1262-Kappa-chain**

DIQLTQSPSFLSASVGDRVTITCWASQGINSYLAWYQQKPGKTPKLLIYAASTLQSGVPSRFSGSGSGTEFTLTISSLQPEDFATYYCQQLNSYPCSFGQGTTLEIK

**>1262-Heavy-chain**

QVQLQESGPGLVKPSGTLSLTCAVSGGSISSSHWWSWVRQPPGKGLEWVGEISLSGSTHYGPSLKSRVSISLDKSMNHFSLRLSSVTAADTAVYYCARESRFYGAYFDYWGQGTLVTVSS

**>1268-Kappa-chain**

DIQLTQSPSFLSASVGDRVTITCWASQGISSFLAWYQQKPGKAPKLLIYAASTLESGVPSRFSGSGSGTEFTLTISSLQPEDFATYYCQQLNSYPRTFGQGTKVEIK

**>1268-Heavy-chain**

EVQLVESGGGLVKPGGSLRLSCAASGFTFSRYSMNWVRQAPGKGLEWVSSISSRSSYKYYVDSVKGRFTISRDNAKNSLYLQMNSLRAEDTAVYYCARDDYGSGSYYYNWLDPWGQGTLVTVSS

**>1269-Kappa-chain**

EIVLTQSPGTLSLSPGERATLSCRTSQSVSSSYLAWYQQKPGQAPRLLMYGASSRATGIPDRFSGSGSGTDFTLTISRLEPEDFAVYYCQQYGNSFGQGTRLEIK

**>1269-Heavy-chain**

EVQLVESGGGLVQSGGSLRLSCAASGFTFSRNAMNWVRQAPGKGLEWVSTISGSGDSTYYADSVKGRFTISRDNSKNTLYLQVNSLRAEDTAVYYCAKGDYYFDSGSYSFGMDVWGQGTTVTVSS
